# Development of a morphology-based modeling technique for tracking solid-body displacements: examining the reliability of a potential MRI-only approach for joint kinematics assessment

**DOI:** 10.1186/s12880-016-0140-1

**Published:** 2016-05-18

**Authors:** Niladri K. Mahato, Stephane Montuelle, John Cotton, Susan Williams, James Thomas, Brian Clark

**Affiliations:** Ohio Musculoskeletal and Neurological Institute, Ohio University, Athens, OH 45701 USA; Department of Biomedical Sciences, Ohio University, Athens, OH 45701 USA; Department of Mechanical Engineering, Ohio University, Athens, OH 45701 USA; School of Rehabilitation and Communication Sciences, Ohio University, Athens, OH 45701 USA; Department of Geriatric Medicine, Ohio University, Athens, OH 45701 USA

**Keywords:** MRI, Stereophotogrammetry, Scientific rotoscoping, Dynamic sequence, Back pain

## Abstract

**Background:**

Single or biplanar video radiography and Roentgen stereophotogrammetry (RSA) techniques used for the assessment of *in-vivo* joint kinematics involves application of ionizing radiation, which is a limitation for clinical research involving human subjects. To overcome this limitation, our long-term goal is to develop a magnetic resonance imaging (MRI)-only, three dimensional (3-D) modeling technique that permits dynamic imaging of joint motion in humans. Here, we present our initial findings, as well as reliability data, for an MRI-only protocol and modeling technique.

**Methods:**

We developed a morphology-based motion-analysis technique that uses MRI of custom-built solid-body objects to animate and quantify experimental displacements between them. The technique involved four major steps. First, the imaging volume was calibrated using a custom-built grid. Second, 3-D models were segmented from axial scans of two custom-built solid-body cubes. Third, these cubes were positioned at pre-determined relative displacements (translation and rotation) in the magnetic resonance coil and scanned with a T_1_ and a fast contrast-enhanced pulse sequences. The digital imaging and communications in medicine (DICOM) images were then processed for animation. The fourth step involved importing these processed images into an animation software, where they were displayed as background scenes. In the same step, 3-D models of the cubes were imported into the animation software, where the user manipulated the models to match their outlines in the scene (rotoscoping) and registered the models into an anatomical joint system. Measurements of displacements obtained from two different rotoscoping sessions were tested for reliability using coefficient of variations (CV), intraclass correlation coefficients (ICC), Bland-Altman plots, and Limits of Agreement analyses.

**Results:**

Between-session reliability was high for both the T_1_ and the contrast-enhanced sequences. Specifically, the average CVs for translation were 4.31 % and 5.26 % for the two pulse sequences, respectively, while the ICCs were 0.99 for both. For rotation measures, the CVs were 3.19 % and 2.44 % for the two pulse sequences with the ICCs being 0.98 and 0.97, respectively. A novel biplanar imaging approach also yielded high reliability with mean CVs of 2.66 % and 3.39 % for translation in the x- and z-planes, respectively, and ICCs of 0.97 in both planes.

**Conclusions:**

This work provides basic proof-of-concept for a reliable marker-less non-ionizing-radiation-based quasi-dynamic motion quantification technique that can potentially be developed into a tool for real-time joint kinematics analysis.

## Background

Visualization of skeletal elements is central to three-dimensional (3-D) kinematic analysis of joint motion. Indirect methods based on tracking surface landmarks (using reflective markers attached to the skin surface) within a calibrated volume (stereophotogrammetry) can contain artifacts (errors of transformation) due to integumentary displacements relative to actual skeletal motion [1–5]. Direct visualization of bony elements during joint motion are typically accomplished *via* fluoroscopy or cineradiography. Unfortunately, both of these techniques require the use of ionizing radiation, and outcomes from these techniques are restricted mostly to two-dimensional (2-D) analyses as the majority of these systems use single-plane imaging [6, 7]. Emergence of the roentgen stereophotogrammetry (RSA) technique has enabled *in-vivo* measurement of complex 3-D skeletal kinematics from a series of radiographs acquired with biplanar, orthogonal fluoroscopy [1, 8, 9]. Although this technique is accurate, it commonly requires surgical implantation of markers in bones [2, 9–12], although model-based RSA techniques have recently begun to appear in the literature [13–15].

Recording a series of joint-motion images using x-ray fluoroscopy and then manually superimposing 3-D models of the same skeletal elements to match corresponding outlines in the x-ray images has been used to quantify *in-vivo* joint motion. [7, 16–18]. More recently, Gatesy *et al*. reported using the scientific rotoscoping (SR) motion analysis technique, which involves biplanar fluoroscopy to image skeletal movements, creation of 3-D models of joint skeleton from high-resolution computed tomography (CT) scans, followed by model-to-image matching and registration (rotoscoping) performed over several frames of images yielding skeletal motion animation and 3-D kinematic data [6, 19–21]. SR was developed from the X-Ray Reconstruction of Moving Morphology (XROMM) motion quantification technique, which tracks implanted markers digitized in biplanar fluoroscopic images captured within a calibrated imaging volume, instead of utilizing the model superimposition technique [6]. Though accurate, both SR and XROMM techniques require corrections of geometrical distortions in images prior to the animation [11, 12, 22–24]. While x-ray-based motion analysis techniques like SR, XROMM, and RSA are clearly novel and advanced, their translation to clinical research (i.e., human subjects research) has been limited due to health-related concerns associated with the radiation exposure [25–29].

From a clinical research perspective, understanding *in vivo* skeletal motion is of interest to both scientists and clinicians [30–32]. More specifically, x-ray-based diagnostic imaging techniques measuring human inter-vertebral displacements have focused mostly on imaging the spine at static end-of-range positions [32–40]. However, qualitative and quantitative assessments of spinal motion have been enhanced by quantitative radiographic techniques that track displacements of pre-assigned coordinate points of specific anatomic locations on orthographic spinal images and by real-time joint-motion evaluation with XROMM-like techniques (using CT/magnetic-resonance-imaging-based 3-D models) and RSA (with per-operative implanted vertebral markers) in human subjects [22, 28, 38, 41–47]. Regrettably, these approaches still require exposure to ionizing radiation and, at times, require marker implantation on the bones.

Magnetic resonance imaging (MRI), when used for quantifying inter-vertebral motion, has mostly been restricted to the analysis of end-of-range sagittal-plane displacements [48–50]. However, dynamic cine-phase contrast (cine-PC) or fast-phase contrast (fast-PC) imaging with ultra-fast gradient echo sequences have been employed for evaluating joint kinematics, especially in ankle, knee, or shoulder motion [51–57]. The main approach for these techniques has been the use of pulse sequences that permit volume extraction from full 3-D motion datasets at selected time points along the range of motion (ROM). However, these techniques can be time-consuming. Additionally, the use of cine-PC sequences require a repeated, cyclic, velocity-controlled motion to be performed at the joint of interest during scanning to make the motion synchronized with velocity-encoded motion capture [57, 58]. Also, these images have low resolution and may present motion artifacts [56, 58, 59]. More recently, the combined use of segmented 3-D anatomical models (from high resolution, ~15 mins duration, static axial scans) registered to low resolution, volumetric images acquired at different joint positions using high speed (~40 sec) T_1_ sequences has been reported [57]. Although such techniques acquire multi-position data with much greater speed, the segmentation of these low-resolution images still require multi-slice images of the experimental quasi-dynamic joint positions. Accordingly, recent advancements in these methods have focused on the acquisition of faster and fewer slices of joint motion (without compromising image resolution) for model-to-image registration and without reducing the accuracy of the technique (time-accuracy tradeoff).

Currently, no modeling techniques exist for quantification of inter-vertebral joint displacements using single-plane or orthogonal magnetic-resonance (MR) image templates for 3-D model registration. Accordingly, our long-term goal is to develop a 3-D model-based technique that permits fast dynamic MR imaging of the human lumbar spine using an open-bore weight-bearing musculoskeletal MRI. Our study focuses on the lumbar spine as low back pain (LBP) is one of the most common reasons for seeking medical care world-wide and accounts for over 3.7 million physician visits per year in the United States alone [60–64]. As such, LBP is arguably one of the most debilitating and costly health disorders, and the development of technologies to aid scientists and clinicians in better understanding the etiology of LBP—as well as in monitoring the effects of therapeutic interventions— is desperately needed.

As a first step towards our long-term goal, we present in this article our initial research and development findings for an MRI-only protocol involving imaging (using a standard T_1_ and a fast contrast-enhanced MRI sequences), a series of pre-determined displacements between solid-body models, 3-D models (segmenting T_1_ weighted axial scans), and a morphology-based rotoscoping strategy for animation and quantification of the displacements. The use of the contrast-enhanced sequence will allow us, firstly, to test the feasibility and reliability of its use as a fast imaging tool and secondly, to compare its outcome with that of the standard high-resolution T_1_ images. The feasibility and reliability of this MRI-based technique is discussed here, and we anticipate further developing this technique into a motion-assessment tool for the lumbar spine and other di-arthrodial joints.

## Methods

### General overview of the experimental design

**Fig. 1:**
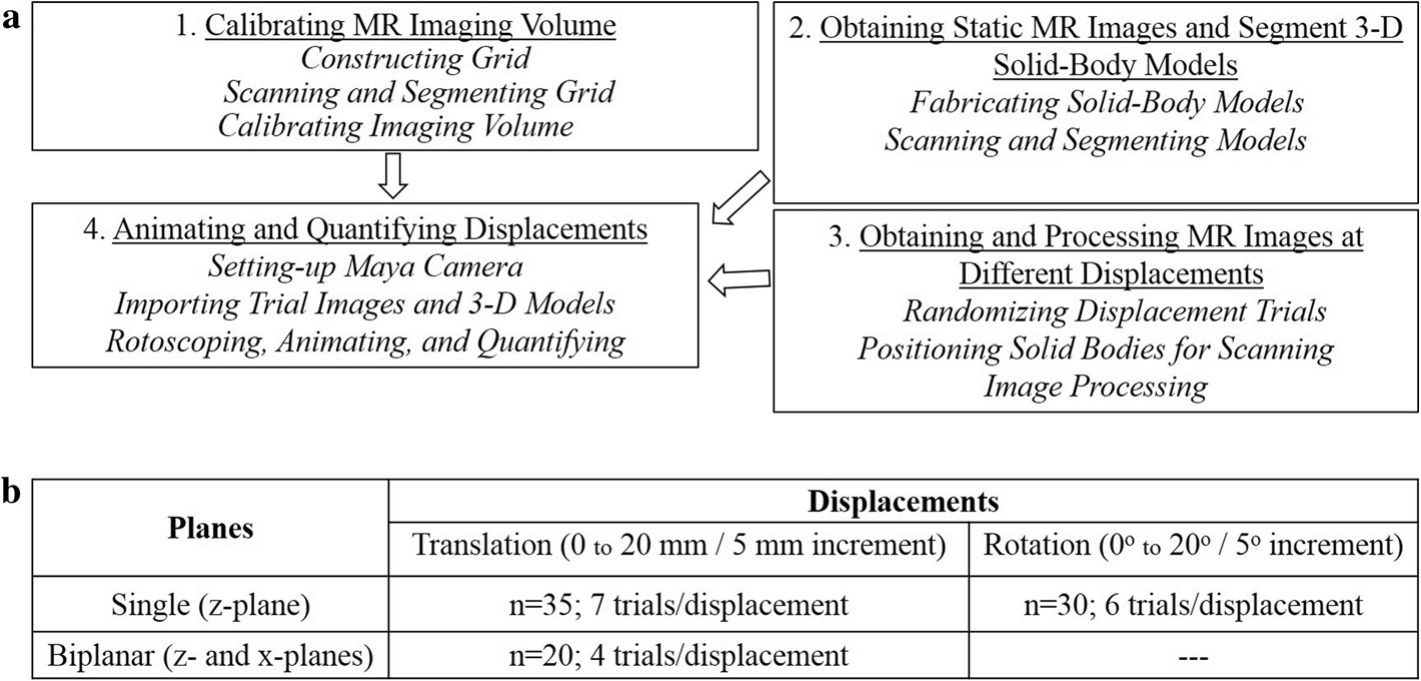
Steps involved in the technique and types of displacements quantified. **a** Overview of the quantification technique. **b** Number of trials for each type of displacement performed. Note that for each displacement paradigm, data from two different pulse sequences were obtained

**Fig. 2:**
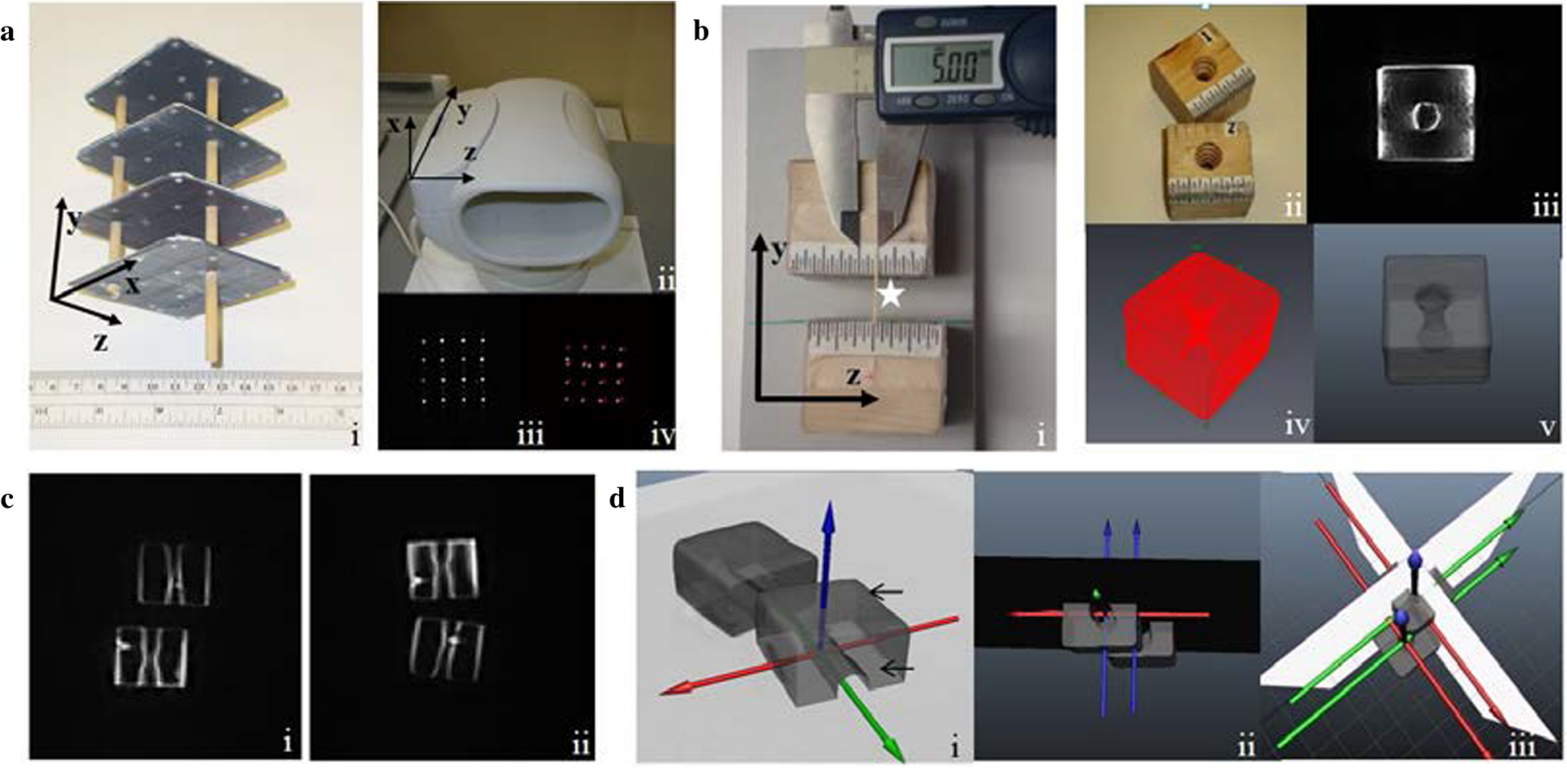
Overview of the animation processes leading to the quantification of a single-plane and biplanar displacements. **a** Imaging volume calibration: (i) the calibration grid with orientation of the plates in space, (ii) MRI coil with orientation of the imaging volume, (iii) & (iv) pre- and post-digitized bead images from the grid. **b** (i) Shows positioning of a translation trial. The solid-body models are spaced ~10 mm apart flat on a foam platform. The lower cube has been translated by 0.5 mm to the right relative to the upper cube, indicated by a wooden pointer (*asterisk*) and measured by the caliper. The orientation of the displacement has been shown by the coordinate axis. (ii) View of the wooden cubes. (iii) High-resolution axial T_1_ image slice through a cube. (iv) Representative 3-D model of a segmented cube. (v) Model as viewed after being imported into the animation environment. **c** (i) Representative image from a single-plane translation trial with the T_1_ sequence. (ii) Representative image from a single-plane rotation trial with the contrast-enhanced sequence. **d** (i) Representative single-plane rotoscoping “scene.” The image slice (off-white background) lies obliquely across the figure. The solid-body shadow is visible with its outline in the image slice (*lower arrow*). Upper half of the superimposed cube model is visible (*upper arrow*) with the anatomical axis. (ii) Image frame from a translation trial viewed from the top of the animation scene. The two cube models are cut through by the image slice (dark horizontal plane) across the hourglass holes within the models. (iii) Orthogonal image slices with registered 3D models

The experiment involved scanning a pair of wooden cubes placed at pre-determined positions (displacement trials) relative to each other in a custom-calibrated coil of an open-MRI system (0.3 Tesla; Esaote G-scan Brio, Genoa, Italy). Additional axial images of the solid cubes were acquired and segmented using the AVIZO software (Hillsboro, OR, USA) to create 3-D virtual models of the cubes. Next, the MR images of the displacement trials and the 3-D cube models were transferred into an animation software (AutoDesk MAYA, San Rafael, CA, USA); and animations of these displacement trials were performed to quantify the relative motion incurred by the solid bodies. The technique involved four principal steps (Fig. 1a). First, the imaging volume of the MRI coil was calibrated using a custom-built grid (Fig. 2a). Second, 3-D models were segmented out from axial scans of the solid-body cubes (Fig. 2b ii-v). Third, the solid bodies were positioned at pre-determined displacements relative to each other in the MRI coil and scanned (Fig. 2b i); and the digital imaging and communications in medicine (DICOM) images were pre-processed into gray-scale TIF format. Fourth, these images were imported into the animation software using calibration data acquired from the grid used in the first step. These images were displayed as a series of background scenes in the animation environment (Fig. 2c & d i-iii). Next, the 3-D models were imported into the animation software and manually manipulated by the user to “register” the models to their outlines visible in the background images (Fig. 2c & d). Lastly, inter-cube translational and rotational displacements were calculated using this technique. All measurements required for fabricating the grid and solid-body cubes and for measuring the experimental displacements during scanning were performed by a digital caliper (sensitivity = 0.02 mm) (Global Industrial, Port Washington, NY, USA). The details of each step are described below.

1. Calibrating the MR Imaging Volume: The volume of the MRI coil was calibrated using a custom-built calibration grid (Fig. 2a). Four square Perspex fiber plates (area = 80 mm^2^; thickness = 2 mm) (Modular-Movement Tray-Set, Games Workshop/NG, UK) were serially stacked with a distance of 30 mm between each plate with three wooden dowels drilled across the plates and glued at all their contact points for stability. Before fixing the dowels, sixteen holes, each 2 mm in diameter, were drilled into each plate in a 4X4 array. Adjacent holes were drilled 20 mm apart from each other. Each hole was fitted with a 2-mm-diameter water bead using a small amount of glue. Three additional beads were embedded into two adjacent plates to define x, y, and z coordinates of the grid (Fig. 2a) [65]. The x- and z-axes were located in the same plane representing the plane of the grid plates, whereas the y-axis extended perpendicular to the plane of grid plates (Fig. 2a). These coordinates were assigned as per the joint coordinate system (JCS) defined by the Standardization and Terminology Committee of the International Society of Biomechanics for studying inter-vertebral motion [65]. To facilitate visualization of the beads in the MR images, the grid was submerged in a 1 % saline solution for 30 s and then air dried for 2 min prior to scanning. The y-axis of the grid was placed along the longer axis of the MRI coil bore (DPA Wrist Coil, Esaote, Genoa, Italy). Four non-contiguous axial 3-mm-thick slices were acquired parallel to and across the grid in a way that each slice image included a single plate with all the 16 beads of a plate in view using a Fast Spin Echo T_2_ sequence (repetition time [TR] = 7810 ms, time to echo [TE] = 120 ms, field of view [FOV] = 200 x 200, Matrix = 256 × 256; resolution = 0.78 mm; voxel dimension = 1.82 mm^3^). The four DICOM files were then transferred to the AVIZO software, where all the beads were segmented and images of all segmented individual plates were stored in the TIF format using Photoshop software (Adobe Systems Inc., San Jose, California) for later use in Step 4. Additionally, the surface rendition of the segmented beads representing a composite view of the entire grid was saved as an .OBJ file for digitization in Step 4.
2. Constructing and Segmenting the Solid-Body Cubes: Two solid-body cubes, with sides measuring ~40 mm, were cut from a wood block (Fig. 2b). Hourglass shaped holes (7-mm base diameters) were drilled through the center of both cubes with a stepped-cone drill. These holes were drilled to create a distinct morphological feature within the cubes and to facilitate the rotoscoping and model-to-scene matching process in a later step. The cubes measured close to the average transverse dimensions of the first lumbar vertebral body in humans, and the hourglass feature simulated the appearance of the vertebral canal in a motion segment [19, 66–68]. Adjacent edges of the cubes were marked with a 20-mm scale with 1.0 mm graduations (Fig. 2b). A neutral position was defined as zero displacement between the mid-lines of the scales. The relative positions between these mid-lines on the scales were manipulated by the user to perform the translation trials with a range of 20 mm in either direction of the neutral position. The opposite sides of the cubes were marked with a protractor to measure inter-cube rotations on both sides of a 0° neutral position at increments of 5° through 90° of rotational displacement. Additionally, 3-D cube models were manually segmented in AVIZO using contiguous high resolution (pixel = 0.78 mm) axial T_1_ weighted scans (TR = 810 ms, TE = 30 ms, FOV = 200 × 200, Matrix = 256 × 256) (Fig. 2b).
3. Displacement Trials: The solid-body cubes were immersed in ~1 % saline for 30 s, wiped dry, and positioned within the MR coil. The long axes of the hourglass-shaped holes in both the cubes were placed along the y-axis of the imaging volume and scanned in the neutral position. The single-plane translations and rotations were performed in the z-plane of the imaging volume. The axis for the rotation trials was formed by the x-plane. The cubes were placed and fixed by double-sided tape on a flat foam platform in the coil to avoid shifting during scans. After scanning the neutral position, the platform was pulled out of the coil; and the cubes were re-positioned for the next trail, with the displacements verified by the Vernier caliper before the platform was re-positioned inside the MRI coil (Fig 2b). A gap of ~10 mm was maintained between adjacent edges of the cubes during translation, a distance representing the average dimension of a human lumbar disc space [69]. For the rotation trials, the center of rotation (COR) of the rotating cube was kept 50 mm away from the center of the stationary cube. A high-resolution (0.78 mm) T_1_ weighted sequence (TR = 810 ms, TE = 30 ms, FOV = 220 × 220, Matrix = 256 × 256, slices = 3, gap = 0, thickness = 5 mm, scan time = ~2 mins/scan) and a fast contrast-enhanced streaming sequence with resolution 0.98 mm (2D hybrid contrast enhanced streaming sequence [2D HYCE S]; thickness = 8 mm, slice = 1, scan time = ~10 s/scan) were used to acquire single-slice images of displaced positions in the mid-sagittal (zy-) plane with the central core of both cubes in view (Fig. 2c). The trials included translations between 0.0–20 mm in 5 mm increments (*n* = 35 trials; 7 trials/displacement) and rotations ranging between 0^o^ to 20^o^ in 5^o^ increments (*n* = 30 trials; 6 trials/displacement) on both sides of the neutral position (Table 1). Biplanar translations were scanned in static positions after the cubes were displaced both in the z- and x-planes through a range of 5 mm increments in a 0.0 to 20 mm range (*n* = 20; 4 trials/displacement). All trials were number-coded and randomly performed in three separate scanning sessions each designated for translational, rotational, and biplanar trials, respectively. For the biplanar trials, additional orthogonal slices were acquired with the central parts of both cubes in view.
4. Animation of the Imaging Volume and Quantification in MAYA [6, 21, 70]: The MAYA software was used to create the animation environment. The environment essentially represented the calibrated MR imaging volume. The software also provided a “camera-view” for the user to view the cube models and the background scene in the calibrated animation environment (Fig. 2d). The user manually manipulated the 3-D models to match and register them to their outlines seen in the background scenes. The steps for creating the animation environment are as follows:
  - Creating a MAYA framespec File [6]:
    The composite grid .OBJ file created in Step 1 was transferred to MAYA, all the beads were serially numbered according to their actual positions in the grid system, and the centroid points for each segmented bead was calculated by the program. Next, the values of the coordinate points for each bead centroid were calculated in the context of all other beads, representing the entire grid volume. The x, y, and z values of all bead coordinate points were merged together to generate the MAYA ‘framespec’ file to be used for the next step of grid digitization.
  - Digitizing the Beads:
    A MEL-script (MAYA Embedded Language-script) command was run in Matlab. An image of a grid plate previously segmented in AVIZO and stored in a TIF format in Step 1 was opened using the Matlab program, and all beads in the plate were digitized serially by clicking over their central points. Next, the framespec file created in the previous step was loaded into the program to yield the Direct Linear Transformation (DLT) coefficient values for the concerned plate [6, 21]. All four plates were digitized sequentially to generate the DLT coefficient value for each plate image. The program allowed automated corrections for minimization of errors and to contain coefficient values ≤ 1 [21]. This step was repeated for all four plates, and each step yielded a plate coefficient value and a “xyz point” .csv file specific to the concerned digitized plate. The data points of the xyz-point files from all the four plates were collated to generate a common “four-plate xyz point” .csv file for the grid [65]. Next, the four-plate xyz-point and the framespec files were loaded into the Matlab program using the MEL-script. One of the segmented plate-images were opened in the Matlab and re-digitized. The four-plate xyz-point file was loaded into the program, and the MEL-script was re-run to generate a “MayaCam”.csv file that was used to re-create the MR imaging within the animation software and to create the camera-view for the user.
  - Rotoscoping, Animation, and Quantification:
    The animation scene was created using the MayaCam file. After the animation environment was created, the background scene was introduced by importing the TIF format images of the trials into MAYA (Fig. 2c & d). These images were clustered into a series of frames, with each series representing a specific trial type. Next, the two 3-D cube models were imported into MAYA using a scaling factor of 0.1 (from segmentation environment in mm to the animation environment in cm). The models were manipulated with the computer mouse and keyboard functions to achieve maximum geometrically alignment and match between the 3-D model and corresponding image outlines in the background scene. The sharp external boundaries and outlines of the hourglass silhouettes within the solid bodies were utilized to facilitate the model-to-scene match (Fig. 2c & d). This process was called rotoscoping. Once rotoscoping in the first frame of the scene (neutral position in the series) was achieved, an Anatomical Joint Axis (AJX) was assigned to the solid bodies. The image of the background scene was then advanced to the next frame and the rotoscoping repeated; this process was repeated for all remaining trial images. For the biplanar translations, two orthogonal camera views were created to provide background scenes of displacements from two different, the x- and z-plane, perspectives (Fig. 2d). Although the animation software generated solid-body motion data for all rotoscoped image frames in all six degrees of freedom, only applicable single-plane measures were extracted for analysis and reporting. Two sessions (S1 and S2) of rotoscoping and displacement quantification were performed separately for translational, rotational, and biplanar motion by a single observer (NKM). All trials were number coded, and the rater was blinded to the displacement type and the pulse sequence used for the scan. The AJX created in Session 1 was used for rotoscoping in the corresponding Session 2. The approximate time for rotoscoping (Step 4(c)) a series of image frames representing a specific trial type, e.g., a seven-translation series, in this study was ~40 min including matching of the neutral position at the start and extracting displacement data from the series at the end.

**Table 1:** Mean values and standard deviations, between-session average coefficient of variation (CV) and intra-class correlation coefficients (ICC) for the solid-body displacements

		Scanned Displacements	Mean±SD for S1	Mean±SD for S2	Mean CV (%)	ICC (95 % CI)
Single (z-) plane	Translation in T_1_(n=7/displacement)	0.0 mm	0.90±0.64 mm	0.73±0.47 mm	14.63	0.99 (0.98–0.99)
		5.0 mm	5.53±0.32 mm	5.30±0.44 mm	1.07	
		10.0 mm	11.60±0.41 mm	11.30±0.45 mm	2.80	
		15.0 mm	15.01±0.54 mm	15.27±0.52 mm	1.22	
		20.0 mm	20.84±0.43 mm	21.13±0.53 mm	1.81	
	Translation in 2D HYCE S (n=7/displacement)	0.0 mm	1.09±0.69 mm	1.32±0.65 mm	13.50	0.97 (0.98–0.99)
		5.0 mm	5.34±0.75 mm	5.20±0.40 mm	4.68	
		10.0 mm	10.35±0.59 mm	10.97±0.59 mm	4.12	
		15.0 mm	14.70±1.05 mm	15.20±0.60 mm	2.39	
		20.0 mm	19.79±0.72 mm	20.25±0.44 mm	1.61	
	Rotation in T_1_(n=6/displacement)	0°	0.21±0.18°	0.22±0.13°	2.29	0.98 (0.97–0.99)
		5°	5.43±0.77°	4.89±0.64°	7.41	
		10°	10.14±0.95°	10.38±0.75°	1.67	
		15°	14.44±1.22°	15.15±1.72°	3.37	
		20°	20.60±0.59°	20.95±0.64°	1.19	
	Rotation in 2D HYCE S (n=6/displacement)	0°	0.11±0.05°	0.12±0.09°	7.59	0.98 (0.97–0.99)
		5°	5.08±0.11°	5.01±0.06°	1.10	
		10°	10.39±0.17°	10.56±0.35°	1.17	
		15°	14.92±0.35°	15.20±0.21°	1.30	
		20°	20.20±0.63°	20.51±0.42°	1.05	
Biplanar (z- & x-planes)	Translation in 2D HYCE S z-plane (n=4/displacement)	0.0 mm	0.65±0.31 mm	0.92±0.61 mm	3.61	0.97 (0.98–0.99)
		5.0 mm	5.44±0.42 mm	5.03±0.32 mm	6.15	
		10.0 mm	10.54±0.59 mm	11.29±0.61 mm	4.84	
		15.0 mm	14.99±0.31 mm	14.71±0.99 mm	1.32	
		20.0 mm	20.94±0.89 mm	21.39±0.98 mm	1.51	
	Translation in 2D HYCE S x-plane (n=4/displacement)	0.0 mm	0.88±0.52 mm	0.83±0.41 mm	3.95	0.97 (0.98–0.99)
		5.0 mm	5.33±0.33 mm	5.12±0.45 mm	2.80	
		10.0 mm	11.40±0.28 mm	10.95±0.50 mm	2.82	
		15.0 mm	15.06±0.48 mm	15.58±0.40 mm	2.41	
		20.0 mm	20.88±0.58 mm	21.27±0.69 mm	1.32	

SD, standard deviation; CV, coefficient of variation; 95 % CI, lower and upper confidence intervals, S1: Session 1, S2: Session 2

### Statistical analysis

Test–retest reliability for the outcomes involving the T_1_ and 2D HYCE S sequences from the two sessions were determined by coefficient of variation (CV), *t*-test, intraclass correlation coefficients (ICC) (two-way random effects model with a single measure of reliability), and 95 % limits of agreement (LOA) analyses. Variability between the outcomes from a single displacement quantified in two different sessions was analyzed using CV. For example, if a particular displacement was quantified as 11.5 mm in Session 1 and 10.8 mm in Session 2, the CV was calculated as: Standard Deviation of the two sessions divided by the mean of the two sessions times 100. Thus, for this example, the CV = (0.5/11.15) × 100 = 4.44 %. The sessions were performed at an interval of one week. Additionally, we used dependent sample t-tests to compare the values between testing sessions.

The ICC was calculated using a (2, 1) two-way random effects model with a single measure of reliability computed over the variance observed in the two sessions. A (2, 1) model was chosen as it allows the determination of any existing systematic bias. The statistical software SPSS (SPSS Inc., Version 21.0, Chicago, IL, USA) was used to calculate the ICC. The main objective of the statistical analysis was to ascertain the reliability of this technique. The relative reliability was assessed by calculating the ICC, which assesses the reproducibility of a measurement relative to a sample of repeated measurements. The absolute stability of a measure typically defines the contribution of the main-error component in the observed variance. To fully understand the absolute stability or reliability of a measure, it is essential to understand the contribution of different components of the measurement error [71, 72]. Accordingly, the measurement error was broken into two components. The first component was defined as the systematic bias, and the second was termed the random error. The systematic bias denoted the contribution of any learning effect on the part of the assessor in explaining the between-session variability of the data, whereas the random error explained a biological or mechanical effect [72, 73]. The first step was to generate Bland-Altman plots using the between-session means and differences data. The correlation (R^2^) between the absolute differences and the means of the between-session values was calculated to determine the spread of the dependent variable. R^2^ values between 0 and 0.1 represented homoscedasticity, suggesting that there was no correlation between the size of the error and the size of the measured variable. Heteroscedasticity was considered to be present with R^2^ values > 0.1 and indicated that the degree of error increased with increase in the values of the measured variable along the scale, e.g., the error term increased as the technique attempted to measure larger displacements (translation/rotation) in the experiment [71–74]. Finally, the ratio LOA was calculated for verification of the absolute reliability of the measure using the following equation: ratio LOA = [(SDdiffs/AVGmeans) × 1.96] × 100, where *SDdiffs* was defined as the Standard Deviation of the difference of scores (Session 1 and Session 2), *AVGmeans* represented the average of the mean scores (Session 1and Session 2) for each measurement, and the factor 1.96 specified the inclusion of 95 % of observations of the differences in scores. The ratio LOA was interpreted as the highest percentage by which two tests will differ due to measurement error in either the positive or negative direction [72].

## Results

### Summary of results

Descriptive statistics and CV and ICC reliability measures obtained from the T_1_ and 2D HYCE S images for each type of displacement are provided in Table 1. A high degree of between-session reliability was observed for both the T_1_ and the contrast-enhanced dynamic pulse sequences. Specifically, the average CVs for translation were 4.31 % and 5.26 % for the two pulse sequences, respectively, while the ICCs were 0.99 for both sequences. For rotation measures, the CVs were 3.19 % and 2.44 % for the two pulse sequences with the ICCs being 0.98 and 0.97, respectively. A novel biplanar imaging approach also yielded high reliability, with mean CVs of 3.39 % and 2.66 % noted for translation in the z- and x-plane, respectively, along with ICCs of 0.97 in both planes. Additionally, all but one displacement variables showed homoscedastic relationships with the Bland-Altman’s LOA analysis of the between-session measurements and demonstrated a relatively low degree of systematic bias.

### Translation trials

**Fig. 3:**
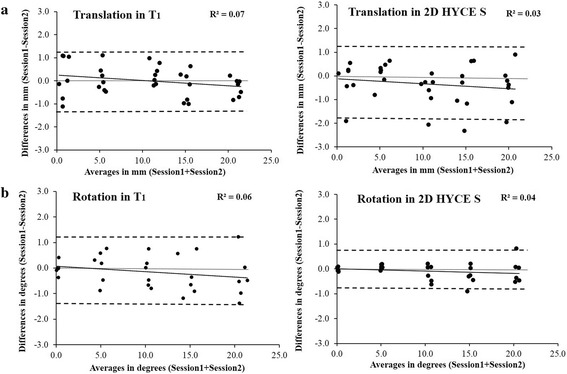
Bland–Altman plots of translation (**a**) and rotation (**b**) trials for each sequence. **a**: Plots of the translation displacements quantified with the two sequences. The dashed lines representing the 95 % confidence interval of test-retest differences for all translations show that the between-session differences were within ±1.24 mm (mean/bias = 0.02 mm) and ±1.59 mm (mean/bias = -0.34 mm) for the T_1_ (left) and the 2D HYCE S (right) sequences, respectively. **b**: Plots of the rotation displacements quantified with the two sequences. The dashed lines representing the 95 % confidence interval of all rotations show that the test-retest differences were within ±1.27° (mean/bias = -0.14°) and ±0.65° (mean/bias = 0.09°) for the T_1_ (left) and the 2D HYCE S (right) sequences, respectively. The central narrow line denotes zero difference mark. The dark line at the center represents the trend line. Homoscedasticity (R^2^ values < 0.1) indicated that the between-session differences in the measurements did not increase with an increase in the magnitude of the measured displacement. Heteroscedasticity was represented by R^2^ values > 0.1, indicating that the between-session differences in the measurements increased with an increase in the magnitude of the measured displacement

Analysis of the between-session measurements of each of the two sequences, applying a paired sample 2-tailed *t*-test, did not show any significant differences in means between the T_1_ (*p* > 0.98) and the 2D HYCE S (*p* > 0.84) pulse sequences. The reliability of the measured variables demonstrated high levels of consistency, with the T_1_ sequence having CVs ranging from 1.1 to 14.63 % and an ICC of 0.99. For the 2D HYCE S sequence, the CVs ranged from 1.6 to 13.5 %, and the ICC was also 0.99. The Bland-Altman plot with 95 % confidence interval (±1.96*standard deviation [SD]) analysis of the between-session data showed that all cases had a test-retest difference within ±1.24 mm (mean/bias = 0.02 mm) and ±1.59 mm (mean/bias = -0.34 mm) for the T_1_ and the 2D HYCE S sequences, respectively (Fig. 3a). The LOA analysis for translation indicated a relatively low degree of systematic bias in the between-session differences (*p* = 0.98 and 0.84) and a homoscedastic relationship between the differences and averages of the between-session measurements for both T_1_ and the 2D HYCE S pulse sequences respectively (R^2^ = 0.07 and R^2^ = 0.03) (Fig. 3a). The homoscedasticity indicated that the random errors did not increase with the increase of the measured displacement values. The follow-up ratio LOA analysis demonstrated a systematic bias in the order of 0.02 and -0.34 and random error of ±14.14 and ±13.68 for the T_1_ and 2D HYCE S sequences, respectively. The ratio LOA analysis for translation suggested that the between-session measurement errors obtained with the technique did not exceed 14.15 % and 13.34 % in either the positive or negative direction with the use of T_1_ and the 2D HYCE S pulse sequences, respectively.

### Rotation trials

Analysis of the between-session measurements of the two sequences applying a paired sample 2-tailed *t*-test did not show any significant mean differences for the T_1_ (*p* > 0.94) and the 2D HYCE S (*p* > 0.96) sequences. The reliability of the measured variables demonstrated high levels of consistency, with the T_1_ sequence having CVs ranging from 1.2 to 7.6 % and an ICC of 0.98. For the 2D HYCE S sequence, the CVs ranged from 1.05 to 7.6 %, and the ICC was 0.98. The Bland-Altman plot with 95 % confidence interval (±1.96*SD) analysis of the between-session data showed that all cases had a test-retest difference within ±1.27° (mean/bias = -0.14°) and ±0.65° (mean/bias = 0.09°) for the T_1_ and the 2D HYCE S sequences, respectively. The LOA analysis for the rotation trials indicated a relatively low degree of systematic bias in the between-session differences (*p* = 0.94 and 0.96) and a homoscedastic relationship between the differences and averages of the between-session measurements for both T_1_ and the 2D HYCE S pulse sequences respectively (R^2^ = 0.06 and 0.04) (Fig. 3b). A homoscedastic relationship indicated that the random errors did not increase with the increase of the measured values. The follow-up ratio LOA analysis indicated a systematic bias in the order of -0.15 and -0.09 and random error of ±14.55 and ±20.10 for the T_1_ and 2D HYCE S sequences, respectively. The ratio LOA analysis for rotation suggested that the between-session measurement errors obtained with the technique did not exceed 14.40 % and 20.01 % in either the positive or negative direction using the T_1_ and the 2D HYCE S pulse sequences, respectively.

### Comparing outcomes between the pulse sequences

**Fig. 4:**
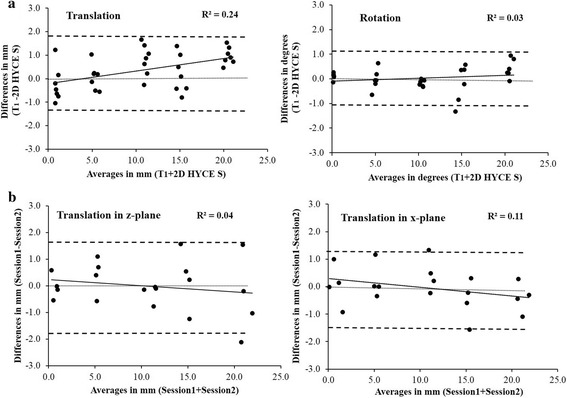
Bland-Altman plots comparing outcomes between T_1_ and the 2D HYCE S sequences (**a**). Plots of the bi-planar translation quantified with the 2D HYCE S sequence (**b**). **a**. Plots comparing outcomes using T_1_ and the 2D HYCE S sequences. The dashed lines representing the 95 % confidence intervals show that the between-session differences in the measurements obtained with the T_1_ and the 2D HYCE S sequences fell within ±1.85 mm (mean/bias = 0.35 mm) for translations (left) and within ±0.95^0^ (mean/bias = 0.02^0^) for all rotations (right) quantified. **b**. Bland–Altman plots for biplanar translations. The dashed lines representing the 95 % confidence intervals show that the test-retest differences for translations fell within ±1.77 mm (mean/bias = -0.01 mm) and ±1.41 mm (mean/bias = -0.04 mm) for the z- and x-planes, respectively. The central narrow line denotes zero difference mark. The dark line at the center represents the trend line. Homoscedasticity (R^2^ values < 0.1) indicated that the random errors did not increase with an increase in the magnitude of the measured values. Homoscedasticity (R^2^ values < 0.1) indicated that the differences in the measurements did not increase with the increase in the magnitude of the measured displacement. Heteroscedasticity was represented by R^2^ values > 0.1, indicating that the differences in the measurements increased with the increase in the magnitude of the measured displacement

Analysis of the difference between the averages of Session 1 and Session 2 translations obtained by T_1_ and 2D HYCE S pulse sequences did not show any significant results using an independent sample 2-tailed *t*-test (*p* = 0.83). The Bland-Altman plot with 95 % confidence interval (±1.96*SD) analysis of the between-sequence data showed a test-retest difference within ±1.50 mm (mean/bias = 0.35 mm) (Fig. 4a). A small heteroscedastic relationship observed in the translation measures indicated that the T_1_ vs 2D HYCE S between-sequence difference in measured translations increased with assessments of larger magnitudes of translation (R^2^ = 0.24). The follow-up ratio LOA analysis demonstrated a systematic bias in the order of 0.354 and random error of ±13.41. The ratio LOA analysis for translation suggested that between-sequence measurement errors were within 13.77 % in either the positive or negative direction.

Analysis of the difference between the averages of Session 1 and Session 2 rotations obtained by T_1_ and 2D HYCE S pulse sequences did not show any significant results using an independent sample 2-tailed *t*-test (*p* = 0.98). The Bland-Altman plot with 95 % confidence interval (±1.96*SD) analysis of the between-sequence data showed a test-retest difference within ±0.95° (mean/bias = 0.02°) (Fig. 4a). A homoscedastic relationship observed in the rotation measures indicated that the between-sequence random errors did not increase with assessments of larger magnitudes of translation (R^2^ = 0.03). The follow-up ratio LOA analysis demonstrated a systematic bias in the order of 0.03 and random error of ±14.28. The LOA ratio analysis for rotation suggested that the between-sequence measurement errors with the T_1_ and 2D HYCE S pulse sequences were within 14.31 % in either the positive or negative direction.

### Biplanar trials

Analysis of the between-session measurements with a paired sample 2-tailed *t*-test did not show any significant difference for the z- (*p* = 0.79) and x (*p* = 0.73) planes. The reliability of the measured variables demonstrated high levels of consistency, with the z-plane having CVs ranging from 1.51 to 6.15 % and an ICC of 0.97. The CVs for the x-plane measurements ranged from 1.32 to 3.95 %, and the ICC was 0.97. The Bland-Altman plot with the 95 % confidence interval (±1.96*SD) analysis of the biplanar between-session data showed that all displacements had a test-retest difference within ±1.41 mm (mean/bias = -0.04 mm) and ±2.70 mm (mean/bias = 0.12) for the x- and z-planes, respectively (Fig. 4b). The LOA analysis for the biplanar trials demonstrated a relatively low degree of systematic bias in the between-session differences (*p* = 0.79 and 0.73) and a systematic bias in the order of -0.04 and -0.01 and random error of ±10.87 and ±21.03 for the z- and for the x-planes, respectively. The ratio LOA analysis for translation suggested that the between-session measurement errors obtained with the technique did not exceed 21.03 % and 10.76 % in either the positive or negative direction for the x- and z-planes, respectively. The ratio LOA analysis for the biplanar translation trials showed a homoscedastic relationship between the differences and averages of the between-session measurements for the z-plane (R^2^ = 0.04), indicating that the random errors did not increase with the increase of the measured values. The x-plane data, however, showed a marginal heteroscedastic relationship (R^2^ = 0.11), indicating that the random errors did marginally increase with the increase of the measured values (Fig. 4b).

## Discussion

In this study, we describe a novel MRI-based approach that is conceptually similar to some fluoroscopy-based modeling protocols with the major difference of not requiring exposure to ionizing radiation, which has obvious implications for clinical research [6, 18, 19]. While this is the first step in the development of an MRI-based protocol of this nature, our initial work indicates that this technique has promise as we have successfully developed a logical and rational approach to the quantification of motion and have also demonstrated relative and absolute reliability. Below we discuss our findings within the context of the extant literature as well as our future directions.

As stated above, the primary innovation of this work is that it represents an MRI-only, morphology-based modeling technique for tracking solid-body displacements, which is similar to fluoroscopy-based approaches, such as RSA, SR, and XROMM, and static x-ray-based techniques. The scope of application of these fluoroscopy-based techniques is limited due to the ionizing radiation exposure. For instance, obtaining serial measures involving significant radiation exposure over time in research studies requiring oversight by an institutional review board (or other analogous committees charged with approving, monitoring and reviewing biomedical research involving humans) could raise questions about the cost-to-benefit ratio, particularly in light of the Institute of Medicine’s recommendation on avoiding unnecessary medical radiation throughout life [26, 28]. Accordingly, we believe that an MRI-only-based modeling technique for investigating joint kinematics has significant advantages, particularly for the advancement of clinical research.

Available MRI modeling techniques have usually applied multi-slice imaging of the objects of interest to capture the experimental displacements introduced into these objects in the scanning environment. Our study has uniquely attempted a morphology-based single-plane and an orthogonal imaging protocol to quantify experimentally induced displacements in our models. Additionally, we have used a fast-scanning protocol with dynamic contrast-enhanced pulse sequence and compared its outcome to a standard high-resolution T_1_ imaging. Both these methods have demonstrated high-levels of reliability in quantifying displacements in objects within the MR imaging volume. These findings provide basic proof-of-concept for the notion that a reliable non-ionizing-radiation-based motion quantification technique can potentially be used to characterize a quasi-static visualization of joint kinematics from a single and biplanar approach. The use of dynamic sequence and image processing can be further explored to attempt quantification of joint kinematics in synchronized motion. Additionally, while our single-plane technique does not objectively address detecting out-of-plane motion, inclusion of the orthogonal imaging in the biplanar approach helps manual positioning of the model to match the corresponding out-of-plane shifts of the image silhouettes.

While our initial development results are promising, our study has some limitations. First, we have used static two-dimensional imaging for quantification purposes; and we do not know whether comparable levels of reliability would have been observed if the dynamic pulse sequence were used to scan the solid-bodies in real-time during an un-synchronized motion with subsequent quantification of these images using the technique reported here. The approach we chose was based on technology currently available; to our knowledge, an MRI-compatible device that would permit real-time manipulation of motion is not commercially available, and the custom development of such a device would require significant resources. Second, the current approach required manual segmentation and post-processing, which is very time intensive. Accordingly, we do not know how the use of semi-automatic protocols or automatic iterative segmentation algorithms would have changed our results. Third, while we reported high levels of reliability for a novel biplanar imaging modality (i.e., quantification of motion in two planes, or coupled motion), the orthogonal images obtained for this analysis were not acquired simultaneously (i.e., an image slice was first acquired in one plane and then acquired for the corresponding orthogonal plane) due to the inherent limitation of MR imaging to do so. While this is not necessarily a limitation of the current work, it could pose a limitation for future work that seeks to acquire simultaneous multi-planar images of motion. Lastly, we only assessed reliability and did not assess accuracy. We are currently conducting experiments that will assess accuracy of our technique in a porcine spine model.

## Conclusion

In summary, this work provides basic proof-of-concept for a reliable non-ionizing-radiation-based marker-less imaging technique that can potentially be used to quantify quasi-dynamic displacements between joint elements. Additionally, this morphology-based MRI-only technique could be explored further as a tool for real-time joint kinematics analysis.

## Abbreviations

RSA: roentgen stereophotogrammetry
MRI: magnetic resonance imaging
2D HYCE S: Two-Dimensional Hybrid Contrast Enhanced Streaming Sequence
CV: coefficient of variation
DICOM: digital imaging and communications in medicine
ICC: intraclass correlation coefficient
LOA: limits of agreement
SR: scientific rotoscoping
XROMM: X-Ray Reconstruction of Moving Morphology
AJX: anatomical joint axis
FOV: field of view
